# Studies on the Effect of Oil and Surfactant on the Formation of Alginate-Based O/W Lidocaine Nanocarriers Using Nanoemulsion Template

**DOI:** 10.3390/pharmaceutics12121223

**Published:** 2020-12-17

**Authors:** Omar Sarheed, Manar Dibi, Kanteti V. R. N. S. Ramesh

**Affiliations:** RAK College of Pharmaceutical Sciences, RAK Medical and Health Sciences University, Ras AlKhaimah 11172, UAE; manar.18909002@rakmhsu.ac.ae (M.D.); venkatramesh@rakmhsu.ac.ae (K.V.R.N.S.R.)

**Keywords:** nanoemulsions, phase inversion temperature (PIT) method, encapsulation, stability, oil type, surfactant concentration

## Abstract

The application of various nanocarrier systems was widely explored in the field of pharmaceuticals to achieve better drug encapsulation and delivery. The aim of this study was to encapsulate lidocaine in alginate-based o/w nanocarriers based on the type of oil (i.e., solid or liquid), using a nanoemulsion template prepared by ultrasound-assisted phase inversion temperature (PIT) approach. The nanoemulsion template was initially prepared by dissolving lidocaine in the oil phase and surfactant and alginate in the aqueous phase, and keeping the PIT at around 85 °C, accompanied by gradual water dilution at 25 °C, to initiate the formation of nanoparticles (o/w) with the aid of low frequency ultrasound. The composition and concentration of the oil phase had a major impact on the particle size and led to an increase in the size of the droplet. The lipids that showed a higher drug solubility also showed higher particle size. On the other hand, increasing the concentration of surfactant decreases the size of the droplet before the concentration of the surfactant exceeds the limit, after which the size of the particle increases due to the aggregates that could be produced from the excess surfactant. The method used produced nanoemulsions that maintained nano-sized droplets < 50 nm, over long-term storage. Our findings are important for the design of nanocarrier systems for the encapsulation of lipophilic molecules.

## 1. Introduction

Nanoemulsions (NEs) are metastable nanocarrier systems comprising a mixture of immiscible liquids in which the dispersed droplets are of average size, between 20 and 500 nm [1]. The system appears to be transparent whereby signs of instability in the formulation becomes apparent in the form of turbidity. It is noticeable that the system is highly susceptible to destabilization, primarily due to the Ostwald ripening [2]; a process that results from the difference in solubility between droplets of different sizes [3]. It occurs due to the mass transport of smaller droplets of the dispersed phase through the continuous phase to reach larger droplets, which then grow in size. In order to achieve a long-term stable formulation that can deliver both hydrophilic and hydrophobic drugs, proper operation with appropriate selection of surfactants and method of preparation is essential [4,5]. Nanoemulsion provides a means to dissolve low solubility drugs, while protecting them from hydrolysis and enzymatic degradation [6].

The small size of the nanoemulsion droplets provides many advantages over other formulations. Droplets can withstand Brownian motion and force of gravity, which plays a major role in physical instability, leading to sedimentation and creaming. It also ensures that the droplets are dispersed uniformly throughout the formulation, preventing flocculation. The surfactant in the formulation adsorbs at the interface to reduce the interfacial tension between the phases. This avoids the possibility of coalescence and maintain kinetic stability. However, there are still some disadvantages to the preparation of nanoemulsions such as the use of special techniques and equipment, with which the user should be well-trained to operate. They could also add to the cost of the preparation, which would make the product less affordable in return [7].

Various methods are used to produce stable nanoemulsions and are classified into high-energy and low-energy methods. The development of nanoemulsions using high energy methods uses strong disruptive forces to break the emulsion of large droplets into new ones of the nanosize scale [8]. One of these methods is ultrasonication, in which a coarse emulsion is prepared and then subjected to ultrasonic waves, mainly at low frequency (20 kHz), which are presented in the form of strong mechanical vibrations. The sonication probe generates high shear forces, hotspots, and turbulence. These in turn, provide cavitation forces that are intense enough to break the droplets of macroemulsions that reduce their size into nano-scale droplets [9].

On the other hand, low-energy methods depend on the energy input produced from chemical potential of the components to form nanoemulsions. Nanoemulsion formation occurs upon the change in the composition or the environment of the system [10]. For example, method of phase-inversion temperature (PIT) is applicable to formulations containing temperature-sensitive surfactants, such as polyoxyethylene-type nonionic surfactants [11]. These surfactants are of an amphiphilic nature and their solubility varies with temperature. If the temperature increases, the polar groups of the molecule dehydrate, making the molecule more lipophilic and thus it changes from being water-soluble into oil-soluble. A point in between these two conditions is at which the solubility of the surfactant in oil and water is somewhat equal. At this particular temperature, the molecule is neither hydrophilic nor lipophilic and is known as the phase inversion temperature [12]. The formulation of nanoemulsion using this technique is a multi-step method. First, a mixture of oil, surfactant and some water are made and stirred at room temperature, which gives rise to a coarse emulsion. This primary emulsion is heated up progressively, until the phase inversion temperature of the surfactant used is reached. The formulation is then cooled by adding water, which ultimately results in the formation of o/w nanoemulsion [13].

Alginates are polysaccharides consisting of linear copolymers of β-(1–4) linked d-mannuronic acid and β-(1–4)-linked l-guluronic acid units [14]. They contain carboxylate functional groups and can easily dissociate in the aqueous phase and give negative charge to the emulsions [15]. The addition procedure of polymers such as alginate or chitosan, and the polymer concentration, might either stabilize or destabilize nanoemulsions [14,16]. It is also thought that application of an external stress such as sonication or microfluidization can lead to modifications on polysaccharides and increase porosity by extending their superficial area [17]. Alginates, as examples of macromolecules, are capable of interacting with surfactants to form coating layers around the oil droplets in emulsions [16]. Therefore, if incorporated in nanoemulsions, it might exert steric or electrostatic repulsions between droplet interfaces, which can be used to counteract the droplets coalescences or gravitational separation [18,19].

This work describes the nanoemulsion prepared by a combination of high- and low-energy methods. Various surfactant-to-oil ratios and different lipids were employed in this study. The resulting nanoemulsions were characterized in order to determine the effect of the formulation variables on the droplet size and the zeta potential. Effects of oil type, oil composition and surfactant composition and the lidocaine effect on particle size was studied. Lidocaine was selected for the preparation of the nanoemulsions and it is an amide-type anesthetic compound [20]. Researchers use lidocaine in a wide range of therapeutic formulations, but, to the best of our knowledge, there are few reports of microemulsions used in local anesthetics, such as pentacaine and tetracaine hydrochloride as dispersed colloidal phase [21,22]. Shukla’s group used a eutectic mixture of lidocaine and prilocaine in the form of microemulsions [23]. Although the aforementioned eutectic mixture can reduce the aqueous solubility of each other, its effect on their combined solubility is small [23]. Our approach aims to use lidocaine solely as prilocaine is associated with methemoglobinemia [24]. Furthermore, we did not come across a work where lidocaine was used alone as a dispersed phase in nanoemulsions or solid lipid nanoparticles, or prepared using the PIT method. The stability of lidocaine NEs, which were surrounded by surfactant-alginate layers, was also evaluated to understand the effect of ultrasound-assisted phase inversion temperature (PIT) method on nanoemulsion properties, upon the incorporation of alginate.

## 2. Materials and Methods

### 2.1. Materials

Lidocaine was donated by Gulf Pharmaceutical Industries (Julphar, UAE). Oleic acid was supplied by Avonchem, (Macclesfield, UK), beeswax by Acros organics, (Geel, Belgium), and coconut oil by LabChem Inc., (Pennsylvania, PA, USA). Tween 80 was purchased from Sigma-Aldrich, (Missouri, MO, USA). Sodium alginate was obtained from Avonchem (Macclesfield, UK). Chemicals for HPLC analysis included water for HPLC, which was obtained from Fisher Scientific, (Loughborough, UK) and acetonitrile and glacial acetic acid which were purchased from VWR Chemicals BDH, (Lutterworth, UK). Cellulose dialysis membrane used for the entrapment study was bought from Samco Silicone Products (Nuneaton, UK).

### 2.2. Solubility of Lidocaine in Lipids

The solubility of lidocaine in the lipids used in the preparation of nanoemulsion was assessed using the method described earlier [25]. A total of 1 mL of each lipid was transferred to a beaker and placed on a hot plate. A total of 50 mg of lidocaine was then added to the lipid and allowed to dissolve. The addition of lidocaine was continued in increments of 50 mg, until the mixture showed signs of crystallization. The mixture was then diluted with a suitable solvent and the amount of lidocaine used was analyzed using HPLC.

### 2.3. Phase Inversion Temperature Measurements

The phase inversion temperature was determined by measuring the turbidity change of the system with temperature change. Measurements were performed using the Litesizer 500 (Anton Paar, Graz, Austria). The samples were kept in quartz cuvette and placed in the measuring chamber. They were subjected to a controlled heating/cooling cycle using the Peltier temperature-control device. The samples were heated from 25 to 90 °C at a rate of 5 °C/min, retained at 90 °C for 10 min, and then cooled from 90 to 25 °C at a rate of 5 °C/min. Turbidity versus temperature curves at 660 nm were plotted.

### 2.4. Preparation of Alginate-Based Lidocaine Nanocarriers Using the Nanoemulsion Template

Lidocaine nanoemulsion was prepared as per the method suggested by Sarheed et al., with some modifications [25]. The nanoemulsion was prepared by combining low-energy method-phase inversion temperature-and high-energy method, ultrasonic homogenization. The formulation was prepared using different oil types at different concentrations, with varying surfactant concentrations. The nanoemulsion formulations were also prepared in the absence of the drug to compare drug formulations with blank formulations.

The nanoemulsion consisted of two phases—the oil phase and the water phase. The water phase was prepared by mixing 25 mL of 0.5% of sodium alginate with Tween 80 at varying amounts; 0.75 g, 1.05 g, and 1.50 g. The mixture was then kept on a magnetic stirrer and heated to 85 °C. The oil phase consists of a mixture of lidocaine and the lipid. The lipids used were oleic acid, coconut oil, and beeswax. A stock formulation of the oil phase was prepared by adding 1.2 g of lidocaine to 0.05 g of oil and heated in order to dissolve the drug in the oil. From this stock, two different amounts of oil phase were weighed; 0.15 g and 0.3 g, and heated. When both phases reached 85 °C, the water phase was added to the oil phase, dropwise, with constant homogenization using the Ultra-Turrax^®^ homogenizer (IKA T25, Staufen, Germany) at 8500 rpm for 5 min. Then, 100 mL of distilled water also heated at 85 °C was added to the mixture with constant homogenization. The resulting concatenations were determined to be 1.2 mg/mL and 2.4 mg/mL. The final concentration of water was kept constant at 97.5% wt.

The resultant nanoemulsion was subjected to ultrasound using a probe sonicator (300 V/T ultrasonic homogenizer, BioLogics Inc., Houston, TX, USA.), at 20 kHz and power 70% for 5 min.

### 2.5. Surfactant Concentration and Oil Type and Composition

A total of 18 nanoemulsion formulations were prepared; 6 for each oil, the amount of surfactant and oil phase for each surfactant-to-oil ratio (SOR) is shown in Table 1. Two groups of NEs were prepared. First, the total lipid content was kept constant, while the SOR was varied by altering the amount of surfactant and vice versa for the second group where the influence of oil content was evaluated. Blank formulations were also prepared using the same method but without lidocaine, whereby the oil phase consisted of the lipid only.

### 2.6. Drug Entrapment Efficiency

Lidocaine entrapment in nanoemulsion was measured by calculating the amount of free drug present in the aqueous phase using the cellulose dialysis membrane method. A cellulose membrane was used with a molecular weight cut-off 3500 Dalton, which was soaked in phosphate buffer solution (PBS) at pH 7.4 overnight, prior to use. A total of 3 mL of the sample formulation was placed in the dialysis membrane and then tightly closed from both sides. The membrane was then immersed in a 100 mL receptor compartment consisting of PBS (pH 7.4) and ethanol, at a ratio of 80:20, to ensure sink conditions. PBS was prepared by mixing 0.5 g of disodium orthophosphate and 0.3 g of potassium dihydrogen phosphate, pH adjusted by pH meter (Sper Scientific Direct, Scottsdale, AZ, USA). The system was covered and placed in a mechanical shaker (Scichem Tech, Bilston, UK) for 24 h. Sample was taken from the receptor compartment and analyzed using HPLC, to determine the amount of free drug that crossed the membrane. Entrapment efficiency was calculated using the following Equation (1):

(1) $$Entrapment\;efficiency=\frac{W_{a}-W_{s}}{W_{a}}$$

*Wa*—amount of drug added to the formulation, *Ws*—amount of unencapsulated drug measured in the supernatant.

### 2.7. Particle Size Measurements

Nanoemulsion droplet size measurement was taken using Litesizer 500 (Anton Paar, Graz, Austria), which uses the dynamic light scattering technique. The samples were measured in standard disposable cuvette, at 25.0 °C and the measurement angle was set on back scatter at an angle of 175°. Droplet size was presented as a mean hydrodynamic diameter. The Stokes–Einstein equation (Equation (2)) was used to calculate the $D_{h}$ as follows:

(2) $$D_{h}=\left(K_{B}T\right)/\left(3\pi \eta D\right)$$

where $D_{h}$ is the particle hydrodynamic diameter, $K_{B}$ is the Boltzmann’s constant, *T* is the absolute temperature, *D* is the translational diffusion coefficient, *ƞ* is the viscosity of the aqueous phase (Pa·s). Each measurement was as a series of 5 repetitions per sample and the mean particle size and standard deviation were determined. The viscosity of 0.5% sodium alginate solution was 24 m Pa·s and was considered to be the viscosity of the dispersant phase during particle size measurements. It was also considered low to affect the DLS measurement. The samples were measured without dilution, as they were very diluted (97.5% wt.) so the effects of multiple scattering could be avoided.

The particle size distribution by number was also determined and a relative refractive index, which is the ratio of refractive index of lipids, oleic acid (1.463), coconut oil (1.430), and beeswax (1.444), to that of the dispersion medium (1.33)—of 1.09, 10.7, and 1.08, respectively, was assumed in the calculation of the particle size distributions. The particle size measurements were also reported as the mean diameters (*d*_43_ and *d*_32_) calculated using Equations (3) and (4) respectively:

(3) $$d_{43}=\;\left(\sum^{\;}n_{i\;}d_{i}^{4}\right)/\left(\sum^{\;}n_{i\;}d_{i}^{3}\right)$$

(4) $$d_{32}=\;\left(\sum^{\;}n_{i\;}d_{i}^{3}\right)/\left(\sum^{\;}n_{i\;}d_{i}^{2}\right)$$

where $n_{i}$ is the number of droplets of diameter $d_{i}$.

Litesizer 500 also offers information about the polydispersity index of the samples, which indicates the breadth of the size distribution.

The polydispersity index (PDI) correlates with the slope of the decay curve. It could be calculated as described in the photon correlation spectroscopy norm (ISO-13321), the cumulant fit is a polynomial fit. The fit function could be written as:

(5) $$y\left(\tau_{i}\right)=a_{0}-a_{1}\tau_{i}+\;a_{2}\tau_{i}^{2}$$

with PDI:

(6) $$\mathrm{PDI}=2a_{2}/a_{1}^{2}$$

Zeta potential was also measured by the Litesizer 500 using electrophoretic light scattering (ELS), which measures the speed of particles in the presence of an electric field. The sample was placed in Omega cuvette, closed with the tips and placed in the measuring chamber. Measurements were made at temperature 25 °C. Each measurement was as a series of 3 repetitions per sample and the mean zeta potential and standard deviation were determined.

### 2.8. Stability Studies

The effect of different surfactant-to-lipid ratios on the NEs stability was studied at room temperature (25 °C), over a period of six months. The dispersions were regularly examined for particle size as well as changes in physical appearance, such as gelation, precipitation, and crystallization.

### 2.9. Quantification of Lidocaine

Lidocaine solubility and entrapment study samples were analyzed by high performance liquid chromatography (HPLC), based on the method reported by the Lee group, with some modifications [26].

The chromatographic column used was Onyx™ monolithic C18, 100 × 4.6 mm, 130 Å, USA. The column temperature was maintained at 25 °C and the volume of injection was 20 μL. The pump used was LC 20AD, Shimadzu, Japan, and the detector was UV visible detector (SPD20A, Shimadzu, Japan). The mobile phase consists of HPLC water and glacial acetic acid, mixed at a ratio of 930:50, and pH was adjusted using 1 N sodium hydroxide to 3.4. Gradient elution was used, in which 4 parts of this solution were allowed to flow through one pump, while 1 part of acetonitrile was allowed to flow through the other. The total mobile phase flow rate was 0.5 mL/min.

The standard stock solution of lidocaine was prepared by accurately weighing 25 mg of lidocaine, dissolving in 3 mL ethanol and making up the volume with water to 25 mL, to obtain a concentration of 1 mg/mL. A series of dilutions were then prepared from the stock solution to obtain solutions of concentrations, 0.05, 0.1, 1, 10, 50, 100, 200, 400, and 600 µg/mL. Chromatograms were integrated at 237 nm and at a retention time between 3.5 and 5.1 min. The calibration curve was plotted between AUC and concentration.

### 2.10. Statistical Analysis

Quantitative data were obtained in triplicates and are reported as mean ± standard deviation. Statistical analysis was performed using Minitab version 19 software. Student’s *t*-test was performed as well. A *p <* 0.05 was considered to be statistically significant.

## 3. Results and Discussion

### 3.1. Lipid Solubility of Lidocaine

**Figure 1:**
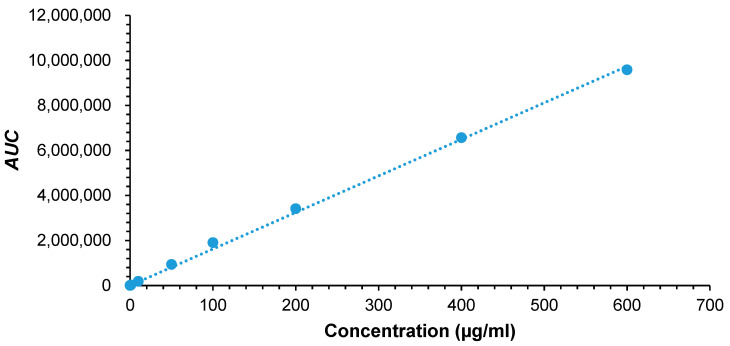
Lidocaine HPLC calibration curve.

The amount of lidocaine solubilized in the hot melted lipids was analyzed by HPLC. For HPLC analysis, a series of dilutions from a stock solution of 1000 µg/mL were prepared to obtain concentrations of 0.05, 0.1, 1, 10, 50, 100, 200, 400, and 600 µg/mL and *AUC* was integrated at 237 nm [26,27]. The calibration curve plotted between *AUC* and concentration, is shown in Figure 1. Lidocaine is a lipophilic molecule with a reported log *P* of 2.44 [28]. Lidocaine solubility was higher in oleic acid (406 mg/mL) than in beeswax (347 mg/mL). Similar results was recently reported by Hamed et al. [29]. While lidocaine’s solubility in coconut oil was found to be the lowest of 64 mg/mL. Oleic acid had a partition coefficient of 7.64, which could provide the highest solubilizing capacity for lidocaine, followed by beeswax and coconut oil [30]. Beeswax has fatty acid esters that give more polar properties compared to oleic acid, so that less lidocaine is solubilized [31]. Whereas, coconut oil is the most polar among other lipids used in this study due to its high lauric acid composition [32]. This limits its ability to dissolve lidocaine. Generally, oil molecules with a small molecular volume or high aromaticity produce a strong solvation effect, resulting in a higher penetration of the oil molecules into the surfactant chain layer, thus improving the rigidity and curvature of the interface [33]. This would also affect the particle size, as various lipids are added.

### 3.2. Phase Inversion Temperature

The phase inversion temperature was identified by measuring the % transmission by Litesizer 500 (Anton Paar, Graz, Austria). The results showed an increase in turbidity when the formulation was heated, which indicated the phase inversion temperature. The PIT was detected at 85 °C, marked by a decrease in the % transmission [25]. Subsequent emulsion cooling reveled a drop in turbidity by an increase in % transmission. This confirmed the transition from w/o emulsion into o/w emulsion [25]. The knowledge of PIT was particularly important during the homogenization, as surfactants could lose their ability to stabilize emulsions if the homogenizer temperature was too close to PIT, due to rapid droplet coalescence [34]. Increased concentration of surfactants had the same effect on the cloud point and was useful in determining the PIT. Based on the results obtained, the PIT process needs to be maintained at constant temperature between 75 and 85 °C, to ensure the formation of bicontinuous microemulsion [25]. At this higher temperature, the interfacial tension decreased and the amount of surfactant adsorbed on the oil–water interface increased gradually, until saturation was achieved [35]. This also guaranteed the formation of stable nanoemulsions, with smaller particles upon cooling and dilution, and the prevention of droplets coalescence.

### 3.3. Preparation of Alginate-Based Lidocaine Nanocarriers from Nanoemulsion Template

Both blank and lidocaine-loaded nanoemulsions were prepared using the low energy method; phase inversion temperature, followed by a high energy method; ultrasonication. Formulations were prepared using three different lipids; oleic acid, coconut oil, and beeswax, and at six different surfactant-to-oil ratios; 5:1, 5:2, 7:1, 7:2, 10:1, and 10:2. Physical appearance of all formulations was noted during the preparation process, after cooling, and throughout the storage period at room and cool temperature. The preparation method used was the same as that proposed previously by Sarheed et al., with modifications [25]. Sodium alginate was added to the formulation in an attempt to produce a nanoemulsion with a controlled release.

In the preparation of the nanoemulsions, Tween 80 and sodium alginate were mixed and heated, where the turbidity of the mixture increased as the temperature reached the phase inversion temperature; 85 °C. As the process continued by mixing the water phase with the oil phase, followed by the dilution, the formulation remained turbid, as the temperature remained at 85 °C. The appearance of turbidity indicated the conversion of the system from o/w to w/o. Keeping the temperature as high as 85 °C, with water dilution, showed that the NEs droplet size would be reduced to 85 nm [36].

Then, each lidocaine formulation was allowed to cool by removing it from the hot plate, allowing it to clear up and the final formulation was a clear and transparent dispersion. The formulations remained transparent both before and after ultrasonication, to help disrupt any droplet aggregates formed during the mixing process Ultra-Turrax^®^ homogenizer. The transparency of the formulation suggests a of small droplet size formulation, which was maintained throughout the storage period. None of the drug formulations showed any signs of instability such as creaming, precipitation, or crystallization. Nor did any of them display any formation of clumps, suggesting that the concentration of sodium alginate was ideal for the physical stability of NEs.

**Figure 2:**
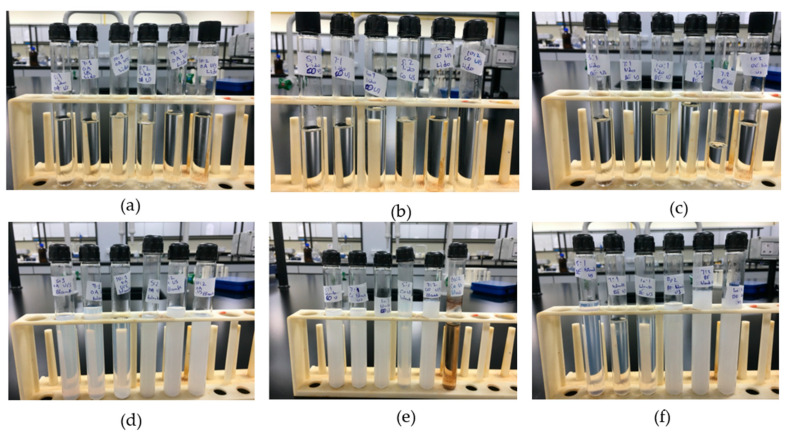
Lidocaine-loaded nanoemulsions with (**a**) oleic acid; (**b**) coconut oil; and (**c**) beeswax. Blank nanoemulsions with (**d**) oleic acid; (**e**) coconut oil; and (**f**) beeswax.

The cooling of the blank formulations showed different results than those of the drug formulations. The turbidity decreased after cooling, but did not reach the transparency of the drug formulations and following ultrasonication, turbidity was further reduced. It is well-known that hydration of the ethylene oxide groups of Tweens would increase significantly by reducing temperature and dilution, and promote the preferred curvature change of the surfactant monolayer, and consequently, the tendency of oil droplet formation [37]. All blank formulations appeared translucent with some showing signs of instability within few weeks. After a duration of 7 months, lidocaine formulations retained a stable transparent appearance, while blank nanoemulsions displayed signs of instability that appeared as creaming. Figure 2 shows all formulated nanoemulsions both lidocaine-loaded and blank. Lidocaine was found to possess surfactant-like properties due to its amphiphilic structure, thus improving lidocaine-containing nanoemulsions stability. This property is discussed later in the study.

### 3.4. Entrapment Efficiency

**Figure 3:**
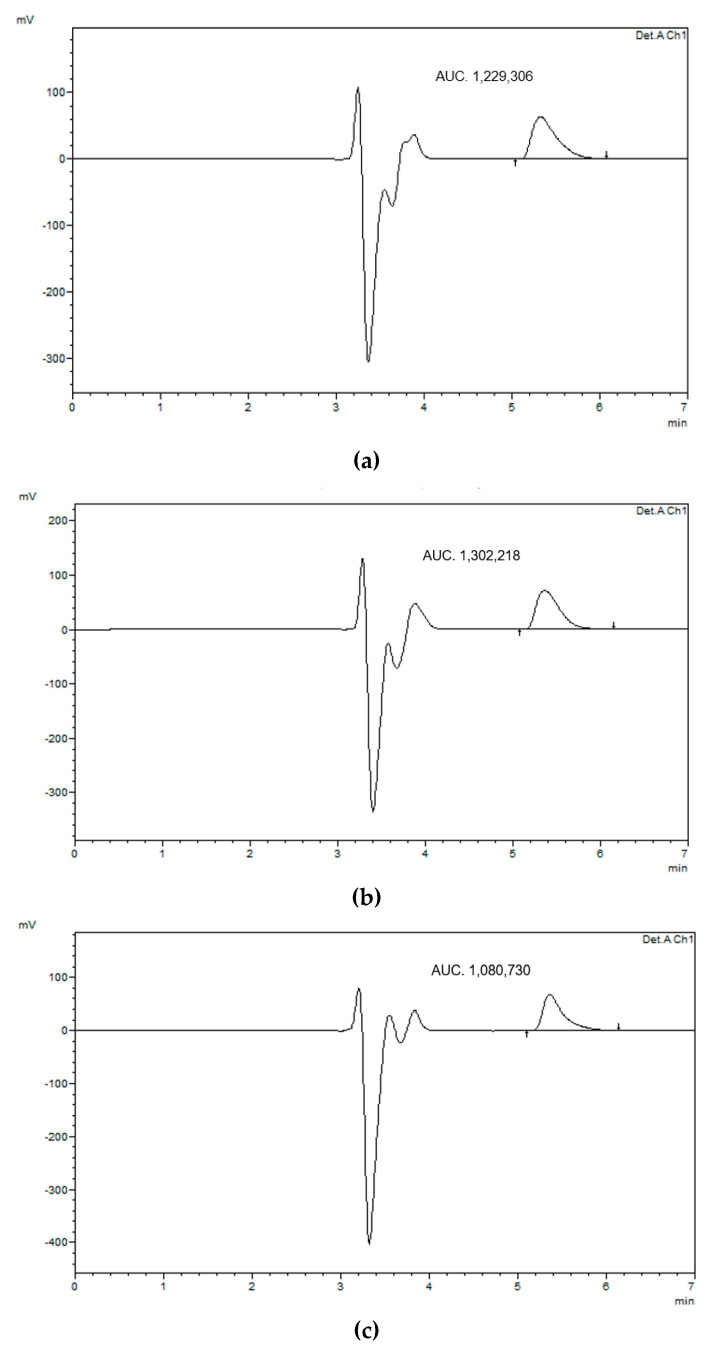
HPLC chromatogram of entrapment study after 24 h of lidocaine-loaded nanoemulsion using (**a**) beeswax, (**b**) coconut oil, and (**c**) oleic acid.

After placing the nanoemulsion formulation in the dialysis membrane for 24 h, the receptor compartment was analyzed using HPLC. Figure 3 shows HPLC chromatograms of the representative formulations prepared at surfactant-to-oil ratio 7:2. It was found that the nanoemulsion formulation was successful in encapsulating the drug within the formulation, with an entrapment efficiency of about 96.9 ± 0.4% for all formulations. This high EE could be attributed to the low surface tension between droplets that prevented their coalescence, which was confirmed by the absence of phase separation and thus enhanced lidocaine solubility and its retention in nanoemulsions [38]. Moreover, lidocaine is a weak base due to the presence of terminal amine group–N-(CH_3_)_2_ that can accept a hydrogen ion and turn it into a positively charged cationic form. This could enable lidocaine to form H-bonds with hydroxyl moieties of Tween 80 and alginate, and thus improve the NEs encapsulation. This behavior could affect the release properties of lidocaine from NEs for various pharmaceutical applications such as transdermal drug delivery. Further studies are therefore needed to assess this effect.

### 3.5. Effect of Surfactant Concentration on Particle Size

**Figure 4:**
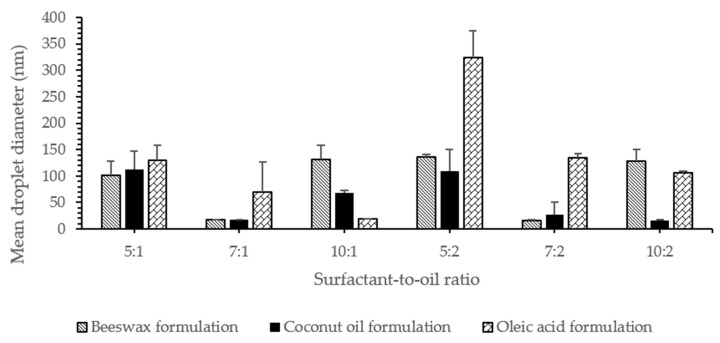
The effect of surfactant concentration on the droplet size of NEs.

The mean droplet diameter for lidocaine nanoemulsion is shown in Figure 4. Results showed that the droplet size of almost all prepared formulations was <140 nm, 39% of which was <60 nm. The mean droplet size of the smaller particles was found to be 15.3, 15.0, and 17.0 nm for nanoemulsions formulated using beeswax, coconut oil, and oleic acid, respectively. The mean size of the larger particles, on the other hand, was found to be 465.7, 517.0, and 534.0 nm. The average hydrodynamic diameter was determined according to the ISO-13321 (1996) and it might lead to misinterpretation in the case of polydisperse systems. In order to have better knowledge of the nanoemulsions, complete particle distribution such as *d*_32_ and *d*_43_ were also reported in this study [34]. This is discussed at a later point in the study.

To identify the relationship between the concentration of surfactant and particle size, nanoemulsions with different surfactant concentrations were prepared and their droplet size was measured. It is worth mentioning that the critical micellar concentration (CMC) of Tween 80 was 0.012–0.015 mM [25]. The surfactant concentration used in this study was between 0.57 to 1.15 mM, which was above the CMC to ensure high drug solubilization and loading, optimal particle size, and long-term stability.

At lower oil concentrations (0.15 g), droplet sizes decreased significantly (*p* < 0.05); 129.1 ± 28.4 nm, 69.4 ± 56.9 nm and 18.8 ± 0.5 nm, with an increase in surfactant concentration, as the surfactant-to-oil ratio was 5:1, 7:1, and 10:1, respectively. This was noticeable when oleic acid was the lipid used and could be due to the surface activity of the surfactant and its solubilization capacity. Surfactants decreased the interfacial tension between the oil and water phases, thus decreasing the amount of free energy required to deform or disrupt the droplets, which resulted in a smaller droplet diameter. They might also form a protective coating around the droplets and prevent them from coalescing one another. However, it is important for the emulsifier molecules to adsorb rapidly enough around the droplets, in order to form this protective interfacial layer [34]. At an SOR of 10:1, the droplet size was the smallest droplet size of 18.8 nm. This might indicate that a monolayer of surfactant was surrounding the oil droplets, taking into account that the length of hydrophilic chain for Tween 80 was 3.8 nm, as stated by Shukla et al. [23,39].

In coconut oil formulations, the droplet size showed an initial decrease (*p* < 0.05) from 112.5 ± 34.2 nm to 17.0 ± 1.0 nm, as the surfactant concentration increased; from SOR 5:1 to 7:1. This could be attributed to the reduction in the interfacial tension resulting from the adsorption of the surfactant molecules on the surface of the oil. The surfactant coverage was adequate to prevent oil droplets from coming close to each other, and thus no coalescence or phase separation was observed in the formulations [40,41]. However, a further increase in surfactant concentration resulted in a significant increase in droplet size to 68.8 ± 4.2 nm, at SOR 10:1. A similar behavior was also observed in beeswax formulations, where the droplet size decreased with an initial increase in the surfactant concentration and increased again (*p* < 0.05) with a further increase in the concentration of the surfactant. This is probably because the amount of surfactant was high enough to initially achieve complete coverage of the oil droplet, along with the presence of excess free surfactant molecules. Excess surfactant molecules might then form aggregates in the continuous phase, which reduces the surfactant concentration available to cover the oil phase, and as a result it would lead to an increase in the droplet size of NEs [40,42]. The presence of excess surfactant could also induce depletion flocculation, as reported by McClements [34]. In addition, the results suggested that there was an optimal concentration of surfactant to formulate a nanoemulsions [33]. This might also indicate that at higher surfactant concentrations, the droplet size was limited by the shear disruptive forces produced by the ultrasound rather than by the amount of surfactant present [43].

At higher oil concentrations (0.3 g), the oleic acid formulation droplet size decreased significantly by 324.1 ± 51.0, 134.3 ± 8.2, and 106.1 ± 2.8 nm, with an increasing surfactant concentration at a surfactant-to-oil ratio 5:2, 7:2, and 10:2, respectively. With coconut oil being the oil used in the formulation, droplet size decreased; 108.9 ± 40.8, 26.5 ± 24.5, 16.7 ± 0.6 nm, with an increased surfactant concentration, with the ratios being 5:2, 7:2, and 10:2, respectively. This similar behavior was also explained by the effect of the surfactant, which adsorbs onto the water–oil interface to reduce interfacial tension, causing droplet disruption and subsequent reduction in droplet size.

Even with an increase in the amount of oil added, with beeswax used as lipid in the formulation, there was still an initial droplet size decrease, followed by an increase. The decrease in droplet size was caused by enough surfactant coverage of oil droplet, while the resulting increase in the droplet size was possibly due to the aggregation of the excess surfactant. Excess of surfactant above the CMC resulted in the formation of micelles with a relatively constant concentration of monomer [34]. These micelles have fairly well-defined average size and shape, under a specified set of conditions and above the CMC, their number appears to increase rather than its size and shape [34].

However, the different concentrations of the surfactant used were high enough to prevent coalescence or any other form of instability in all formulations, where no phase separation was observed.

### 3.6. Effect of Oil Concentration on Particle Size

The increase in the concentration of the oil phase at a specific surfactant concentration also had an effect on the droplet size. The excess amount of oil caused the size of the emulsion droplets to increase [40].

The expected increase in droplet size was detected by particle size measurement, as a result of the increase in the dispersed phase [23]. This behavior was evident in oleic acid formulations. When comparing the surfactant-to-oil ratios, 5:1 and 5:2, it was noted that the droplet size increased significantly from 129.2 ± 28.4 to 324.1 ± 51.0 nm. At ratios 7:1 and 7:2, the droplet size increased (*p* < 0.05) from 89.2 ± 66.0 to 134.3 ± 8.2 nm, and at ratios 10:1 and 10:2, the droplet size changed from 18.8 ± 0.5 to 123.5 ± 39.2 nm significantly.

However, the rise in oil phase concentration did not have any significant effect (*p* > 0.05) on the droplet size in coconut oil formulations. The change was observed only at a higher surfactant concentration, with a change in SOR from 10:1 to 10:2, which showed a significant droplet size decrease (*p* < 0.05) from 68.8 ± 4.2 to 16.74 ± 0.60 nm.

On the other hand, beeswax formulations showed an increase in the droplet size only at a lower surfactant concentration. At an SOR of 5:1, the droplet size was 101.6 ± 26.2 nm, while it was 135.9 ± 4.8 nm at an SOR of 5:2 (*p* < 0.05). At a higher surfactant concentration, the concentration of oil did not seem to have a significant effect on the droplet size as it increased (*p* > 0.05).

### 3.7. Effect of Oil Type

At a specific surfactant-to-oil ratio, changing the lipid used in the formulation had an effect on the droplet size. However, beeswax and coconut oil formulations showed a close relationship in particle size. There was only a significant difference (*p* < 0.05) in the droplet size at a higher surfactant concentration. At the ratio of 5:1, all formulations showed similar droplet sizes, whereas at 7:1, both coconut oil and beeswax formulations had a smaller droplet size (*p* < 0.05) than oleic acid formulation. At 10:1, there were significantly different droplet sizes (*p* < 0.05) in which beeswax displayed the highest, followed by coconut oil and then oleic acid; 109.7 ± 54.1, 68.8 ± 4.2 and 18.8 ± 0.5 nm, respectively.

At ratios of 5:2 and 7:2, beeswax and coconut oil formulations showed a close droplet size (*p* > 0.05), which was smaller than that of oleic acid. At 10:2, however, beeswax again displayed the highest droplet size (128.7 ± 21.4 nm), followed by oleic acid (106.1 ± 2.8 nm) and finally coconut oil (16.7 ± 0.6 nm).

Oleic acid nanoemulsions had the highest particle size in most of the formulations, relative to formulations prepared using other lipids. This could be due to the solubility of lidocaine in oleic acid, which was also the highest. As described earlier, lidocaine was found to be highly soluble in oleic acid, which increased the amount of drug in the oil phase to be solubilized and thus increased the size of the droplets. Shukla also reported the same effect on the particle size and was attributed to the angular structure of oleic acid that could cause larger particles [39]. Leung and Shah [33] found that increasing the oil chain length led to less penetration of oil molecules into the interfacial film, resulting in larger particles, since attractive steric forces predominate. They also concluded that long chain oil is a poor solvent for interfacial film.

Oils with a high concentration of polar compounds were reported to reduce the interfacial tension and facilitate droplet disruption during high pressure homogenization [44]. Oleic acid was considered to be the main non-polar fatty acid in lipids [45]. In this sense, oleic acid was less likely to be solubilized in the aqueous phase, resulting in larger particles [19].

Beeswax and coconut oil nanoemulsions showed smaller droplets relative to oleic acid NEs. Wax esters accounted for 70% of beeswax [31]. These components were fatty acids that were esterified to a fatty acid alcohol, mainly palmitate, palmitoleate, hydroxypalmitate, and oleate [46]. These compounds provided high polarity to beeswax, compared to less polar oleic acid. This would lead to a decrease in the interfacial tension resulting in a smaller particle size.

Coconut oil was mostly composed of lauric acid, accounting for 40% of its constituents, as reported by Rizza’s group [32]. Coconut oil showed the lowest measured contact angle at various cooling temperatures, compared to Jatropha curcas oil and sunflower oil, which are rich in oleic acid and linoleic acid, respectively. This was due to the high polarity of lauric acid relative to oleic acid, which had a weak polarity. In addition, lauric acid had low dipole-generated interactions, resulting from the movement of electrons, which results in low interactions between lauric acid molecules. This reduced the viscosity and thus achieved a low contact angle. [32]. It was also observed that the higher the viscosity of the oil phase, the higher the droplet size, and the more the energy required to disrupt the oil droplets [19,43]. Moreover, the straight chain of lauric acid might also explain the smaller droplets observed in this study with the use of coconut oil.

### 3.8. Effect of the Drug on Particle Size

Upon formulating the drug-loaded nanoemulsion, lidocaine was added to the oil phase, which was then mixed with the water phase, to produce the final dispersion. It was noticed that the addition of drug influenced the behavior of the blank formulation. Generally, lidocaine was found to impart a form of stability to the final formulation. Most of the blank formulations that were as-prepared were considered turbid. While all formulations produced upon the addition of lidocaine were clear nanoemulsions. This could be attributed to the inherent properties of lidocaine. Due to its chemical structure, it was suggested that lidocaine had a surfactant effect. Lidocaine and other anesthetics were identified as amphiphilic in nature. Similar to the surfactants, at a certain concentration they appeared to form micelles; CMC. Uesono et al. showed this surfactant behavior, in which lidocaine and other anesthetics were comparable with traditional surfactants of their ability, to generate an emulsified formulation [47]. Sadurní group also demonstrated an enhancement in the stability of nanoemulsions with addition of lidocaine, as its chemical structure consists of a hydrocarbon chain, an aromatic ring, and an amide group, which imparts the amphiphilic behavior [48]. The effect of lidocaine was explained by Yuan et al. by the fact that lidocaine is polar and it is this polarity that enables lidocaine to interact with the surfactant and the interface linkers, which would increase the hydrophilicity of the oil phase and thus increase the nanoemulsion stabilization [49].

**Figure 5:**
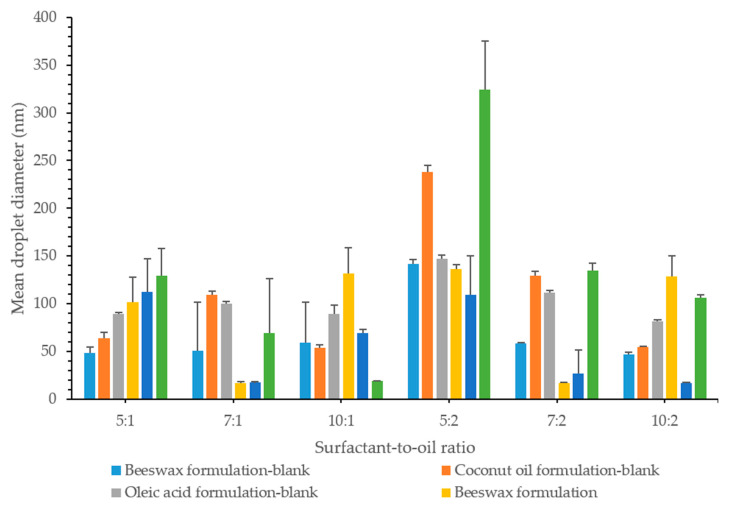
Mean droplet size of blank and drug formulations showing the effect of oil composition, oil type, and the effect of drug.

With regard to the particle size, 50% of the formulations exhibited an increase in the droplet size, upon the addition of lidocaine, relative to blank formulations, as shown in Figure 5. This was not, however, reflected on the physical appearance of the formulation in which they exhibited a continued state of clear stable nanoemulsion, whereas most of the blank formulations showed a turbid appearance. At lower oil concentrations, with a surfactant-to-oil ratio 5:1, all formulations showed a significant increase (*p* < 0.05) in the droplet size. However, a droplet size decrease was observed at 7:1, except for the oleic acid formulation, where no change was observed. At 10:1, with the exception of oleic acid whose droplet size decreased significantly (*p* < 0.05), beeswax and coconut oil formulations showed an increase in the droplet size.

### 3.9. Polydispersity Index

**Figure 6:**
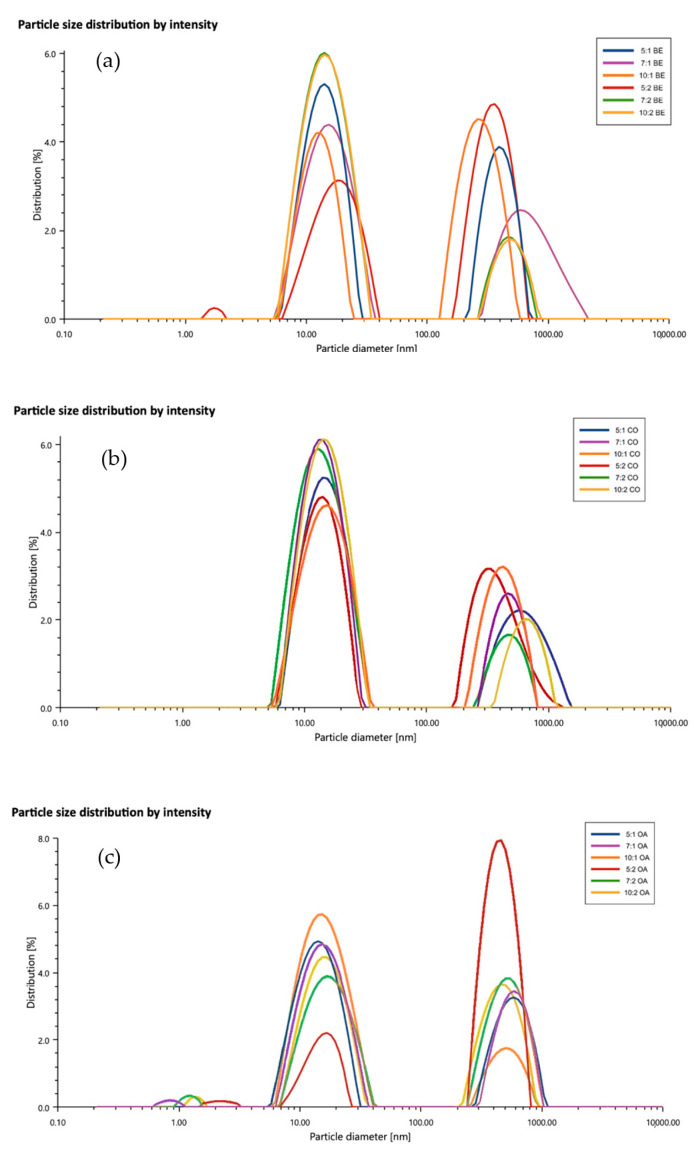
Particle size distribution of lidocaine nanoemulsions. (**a**) beeswax, (**b**) coconut oil. and (**c**) oleic acid formulations.

During the measurement of the particle size, the distribution of the particles in the samples was also measured. The formulated nanoemulsions were found to be of a polydisperse nature, as shown in Figure 6a–c, in which the PDI values fell in the range of 20–30%. It was predicted by Eriksson and Ljunggren [50] using the multiple chemical equilibrium approach that stable microemulsion polydispersities should be in the range of 10–45%. This is based on the assumption that these systems have droplets that are viewed as loosely bonded complex rather than small droplets in the strict sense of the word [50]. This behavior was also reported by other groups and no phase separation was observed [23,39,51,52]. One explanation for high PDIs is the formation of a bimodal distribution, with one population of small droplets around 15.3, 15, and 17 nm for nanoemulsions, formulated using beeswax, coconut oil, and oleic acid, respectively, and another population of large droplets around 465, 517, and 534 nm, respectively. This was similar to the data reported by Mayer et al. [53]. Another explanation for the larger PDI was the overestimation of the cumulant analysis, which represents a small correction to the shape of the correlation function [23]. The mean particle diameter measurements of *d*_32_ and *d*_43_ showed that almost all NEs could be produced with the majority of small droplets in the range of 15–20 nm (Supplementary Material Table S1). On the other hand, the full particle size distribution measurements indicated that some droplet aggregation had occurred, resulting in bimodal distribution. In view of the relatively high concentration of alginate and high zeta potential of the above –60 mV, it could be assumed that high electrostatic and steric repulsion between NEs droplets could occur. Mun et al. [16] postulated that a high concentration of alginate could induce a considerable depletion attraction between the NEs droplets that led to some droplet flocculation (depletion flocculation) during the preparation. Attractive depletion interaction occurred between the emulsion droplets, when they were surrounded by small nonadsorbing colloidal particles, such as surfactant micelles, polymers, or nanoparticles [34].

As discussed earlier, the formation of bimodal distribution could be considered to be the main reason for a high polydispersity index. The presence of alginate in the colloidal systems was proposed to be responsible for the multimodal distribution and high PDIs of nanoemulsions [14,53,54]. Artiga-Artigas et al. studied the effect of sodium alginate incorporation on the particle size distribution of nanoemulsions [14]. They observed a multimodal particle distribution and it was attributed either to unadsorbed surfactant micelles at the oil droplets interface, which were repelled due the presence of excess alginate molecules or due to alginate aggregates. The use of ultrasound was also reported to give multimodal distribution at any amplitude or power, as reported by the Salvia-Trujillo group [54]. However, the use of ultrasound was justified by its ability to disrupt larger droplets and to stabilize the nanoemulsion by creating smaller ones. This was confirmed by the transparent appearance of nanoemulsions and the lack of phase separation in our study. Ultrasound exerts its physical impact through cavitation, which can induce polymer depolymerization. This could further improve the stability of nanoemulsions by reducing the steric impediment of alginate polymer chains during their disposition around the oil droplets [55]. Thus, if the system was not subjected to high shear stress, this could result in the formation of polymer aggregates [14].

Khorasani and Pourmahdian investigated the synthesis of hydrogel nanoparticles through the inverse microemulsion polymerization method and reported that the use of higher amounts of water upon dilution at constant concentration of Tween 80 would lead to the expansion of the continuous phase and significant increase in the interfacial surface area. As a result, the surfactant was no longer able to sustain nanoemulsion stability without changing the particle size and thus the PDI would increase [52].

Another possibility of higher PDIs might be due to the homogenization used to emulsify the water and oil phases [54]. During homogenization, eddies are formed and the fluid around these regions is disrupted and deformed. Normally, eddies of different sizes are formed in the fluid, in which large-sized eddies produce shear stresses that are not very effective in deforming the droplets. On the other hand, small-sized eddies produce high shear stresses that are dissipated in the fluid medium. Only medium-sized eddies are effective in disrupting the droplets. Thus, due to the different size of the eddies present, a polydisperse system is more likely to be formed. In addition, the size of the droplets is determined by the length of time spent in the homogenizer disruption region, which contributes to the creation of a polydisperse system [34]. It was also proposed that both alginate and Tween 80 could adsorb on the lipid surface, leading to the formation of a complex interface that is reflected on the particle size distribution [54]. It was postulated that the driving force of alginate adsorption to the NEs droplets was electrostatic in nature [16].

### 3.10. Zeta Potential

Zeta potential is considered an effective way to describe the surface potential of the suspended droplets. Thus, the electrical properties of the nanoemulsion formulation were measured through obtaining zeta potential values. All formulations showed a negative charge greater than −60 mV, reaching a relatively constant value of between −70 to −80 mV, suggesting that the emulsion droplets reached saturation with alginate rather than Tween 80 [16], as shown in Supplementary Material Figure S1. Zeta potential was measured in triplicates for each formulation and the mean value is shown in Table 2. The measured zeta potential reflected the observed stability of the lidocaine formulations, in which the physical appearance was a clear nanoemulsion with no signs of instability, such as creaming. Generally, a zeta potential greater than ±30 mV is considered adequate to ensure the physical stability of nanoemulsion [56]. High zeta potential value ensures stability because a charge that is sufficiently large, can prevent the aggregation of the droplets due to electrostatic repulsion between the droplets.

The nanoemulsions were found to have a negative charge, because the droplets might have an electrical charge that depends on the types of ionizable molecules present and the pH of the aqueous phase [34]. The charge is explained by the rise in the concentration of Tween 80, which would lead to a reduction in the zeta potential. This occurs because an increase in the concentration of the surfactant above a critical value results in the sudden expulsion of OH-groups from the o/w surface, which can reduce the surface potential and thus the potential for zeta [57]. However, it was reported that Tween 80 was responsible for a slight increase in the negative charge on the oil–water interface [19,58]. Therefore, alginate could be considered the reason for the high zeta potentials in this work. Alginate has carboxylate and hydroxyl functional groups that are easily deprotonated at neutral pHs [14,59]. Furthermore, the application of shear stress, such as ultrasonication, can break up or modify the alginates chain and release more number of free molecules that can be potentially adsorbed on the oil–water interface [60]. As a result, more deprotonated groups around the oil droplets would be deposited and high zeta would be produced, which would be able to stabilize nanoemulsions by preventing re-coalescence [14]. This could support the electrostatic effects on the physical stability of nanoemulsions. Zeta potential data also indicates that the concentration of alginate in NEs was optimal to achieve high net negative charges, which could enable droplets to repel each other electrostatically [16].

### 3.11. Stability Study of Lidocaine NEs

**Figure 7:**
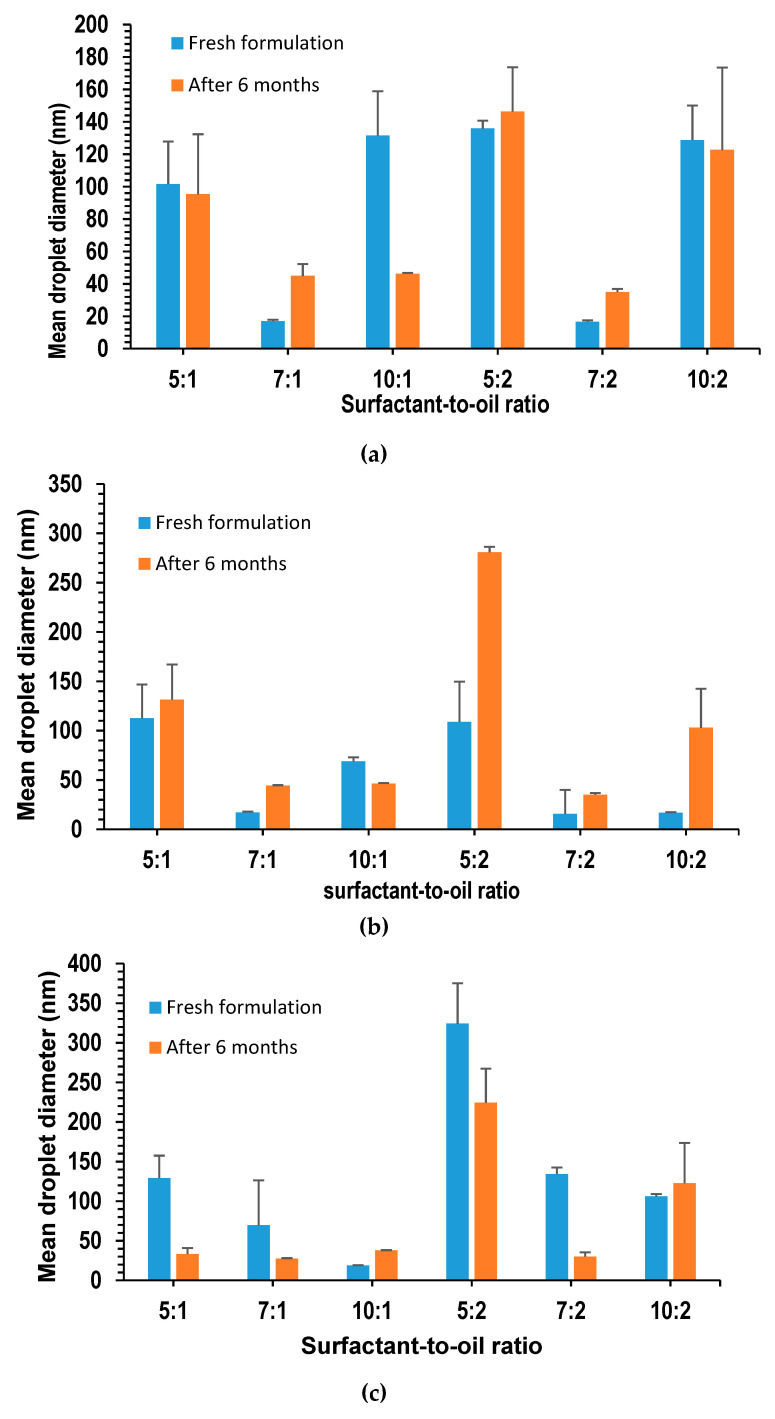
Droplet size of lidocaine-loaded nanoemulsions with (**a**) beeswax; (**b**) coconut oil; (**c**) oleic acid; freshly prepared and after 6 months.

The long-term stability of the nanoemulsions is characterized by both physical observation and measurement of the droplet size, over the entire storage period. On physical inspection, it was shown that the formulation retained its transparent appearance without any signs of creaming or phase separation. The NEs in this study were stable, compared to the one produced by Machado [61], in which phase separation occurred several hours after the preparation. The presence of a double layer of surfactant and polymer around oil droplets was reported to minimize the creaming rate, by controlling the net density of the droplets and bringing it closer to that of the surrounding aqueous phase [16]. The transparency of the formulation indicated a small droplet size that was further illustrated by the particle size measurement. Figure 7a–c display the droplet size measurements of various NEs prepared; it was found that all formulations had a droplet size less than 150 nm. After 30 weeks, 44.4% of the formulations experienced a droplet size decrease, while 55.6% of the formulations were found to have an increased droplet size. The increase in droplet size could be ascribed to the Ostwald ripening, in which droplets of smaller sizes tend to diffuse into larger droplets, due to their higher chemical potential. This was reported to be the most common mechanism for destabilizing nanoemulsions [48,62].

However, even with the measured increase in the droplet size, it remained below 150 nm, with the exception of coconut oil and oleic acid formulations at an SOR of 5:2. However, the increase was not reflected on the physical appearance of the formulations, which as mentioned earlier, remained as clear and transparent as the fresh formulations.

To differentiate between the different mechanisms of instability, the cubes of the average radius *r*^3^ of emulsions were plotted against time, in which a linear relationship was evidence of Ostwald ripening. The Lifshitz–Slezov and Wagner (LSW) theory [48] describes the rate of Ostwald ripening, Equation (7) is as follows:

(7) $$\omega =\frac{dr^{3}}{dt}=\left(8/9\right)\left[\left(C\infty \gamma VmD\right)/\rho RT\right]$$

where C∞ is the bulk phase solubility (the solubility of the oil in an infinitely large droplet), γ is the interfacial tension, *Vm* is the molar volume of the oil, *D* is the diffusion coefficient of the oil in the continuous phase, ρ is the density of the oil, *R* is the gas constant, and *T* is the absolute temperature.

On the other hand, it was suggested that, when a linear relationship is obtained by plotting 1/*r*^2^ against time, it should imply coalescence [48,62]. No such linear relationship was obtained, which might be because neither of these mechanisms is predominant and the two breakdown processes can occur concurrently in this system, as suggested by Sadurní et al. [48].

## 4. Conclusions

In conclusion, a stable lidocaine nanoemulsion was successfully formulated using a combination of high- and low-energy methods; ultrasonication and phase inversion temperature, respectively. The method used produced nanoemulsions that maintained nano-sized droplets <50 nm over long-term storage. Nanoemulsion formulated using Tween 80 as a surfactant at varying surfactant concentrations and using various lipids in the oil phase, oleic acid, beeswax, and coconut oil. The use of lidocaine in the formulation was shown to impart a degree of stability to the formulation due to its relative amphiphilic properties.

It was found that an increase in oil concentration contributed to an increase in the size of the droplet. Increasing surfactant concentration, on the other hand, was shown to decrease the droplet size, as it reduced the interfacial tension and provided a protective cover for the droplets. This effect was observed until the surfactant concentration reached a limit, after which the droplet size increased due to the aggregates that could form from the excess of the surfactant. Lipid is also shown to have an effect on the droplet size that is associated with the drug solubility. The lipid that showed higher drug solubility also showed a higher droplet size. Nanoemulsion formulation was proven to be a promising approach to encapsulating the active pharmaceutical ingredient, lidocaine, to a high extent.

## Figures and Tables

**Table 1:** The composition of lidocaine nanoemulsions (NEs).

Surfactant-to-Oil Ratio	Surfactant Amount (g)	Oil Phase Amount (g)
5:1	0.75	0.15
7:1	1.05	0.15
10:1	1.50	0.15
5:2	0.75	0.30
7:2	1.05	0.30
10:2	1.50	0.30

**Table 2:** Zeta potential of lidocaine NEs.

Surfactant-to-Oil Ratio	Lipid Type	Zeta Potential
5:1	Beeswax	−80.57 mV
	Coconut oil	−71.13 mV
	Oleic acid	−79.56 mV
7:1	Beeswax	−68.08 mV
	Coconut oil	−71.42 mV
	Oleic acid	−76.60 mV
10:1	Beeswax	−71.18 mV
	Coconut oil	−66.68 mV
	Oleic acid	−70.45 mV
5:2	Beeswax	−68.54 mV
	Coconut oil	−76.33 mV
	Oleic acid	−73.82 mV
7:2	Beeswax	−67.29 mV
	Coconut oil	−70.09 mV
	Oleic acid	−71.85 mV
10:2	Beeswax	−71.32 mV
	Coconut oil	−66.47 mV
	Oleic acid	−61.91 mV

## Supplementary Materials

The following are available online at https://www.mdpi.com/1999-4923/12/12/1223/s1, Table S1: Experimental values obtained for *d*_32_ and *d*_43_, Figure S1: Zeta potential of lidocaine-loaded nanoemulsions formulated with (a) beeswax, (b) coconut oil and (c) oleic acid formulations.
