# Supplementary material for: Studies on the Effect of Oil and Surfactant on the Formation of Alginate-Based O/W Lidocaine Nanocarriers Using Nanoemulsion Template
Source: Pharmaceutics. 2020 Dec 17;12(12):1223. doi: 10.3390/pharmaceutics12121223 (PMC7766092; doi:10.3390/pharmaceutics12121223)
Supplement: Supplementary file 1 [file pharmaceutics-12-01223-s001.pdf]

Supplementary Materials: Studies on the effect of oil and surfactant on the formation of alginate-based o/w lidocaine nanocarriers using nanoemulsion template

Omar Sarheed*, Manar Dibi and Kanteti V.R.N.S. Ramesh

Table S1. Experimental values obtained for d_{32} and d_{43}

Surfactant-to-oil ratio	Lipid type	d_{32}	d_{43}
5:1	Beeswax	22.1 nm	37.6 nm
	Coconut oil	111.7 nm	180.9 nm
	Oleic acid	13.2 nm	15.9 nm
7:1	Beeswax	12.0 nm	13.9 nm
	Coconut oil	12.1 nm	13.9 nm
	Oleic acid	13.5 nm	16.1 nm
10:1	Beeswax	13.2 nm	16.3 nm
	Coconut oil	12.3 nm	14.6 nm
	Oleic acid	15.0 nm	17.7 nm
5:2	Beeswax	13.3 nm	16.4 nm
	Coconut oil	12.6 nm	15.3 nm
	Oleic acid	303.6 nm	350.5 nm
7:2	Beeswax	14.2 nm	18.9 nm
	Coconut oil	10.9 nm	12.8 nm
	Oleic acid	17.8 nm	23.3 nm
10:2	Beeswax	14.4 nm	18.4 nm
	Coconut oil	11.7 nm	13.6 nm
	Oleic acid	13.2 nm	15.6 nm

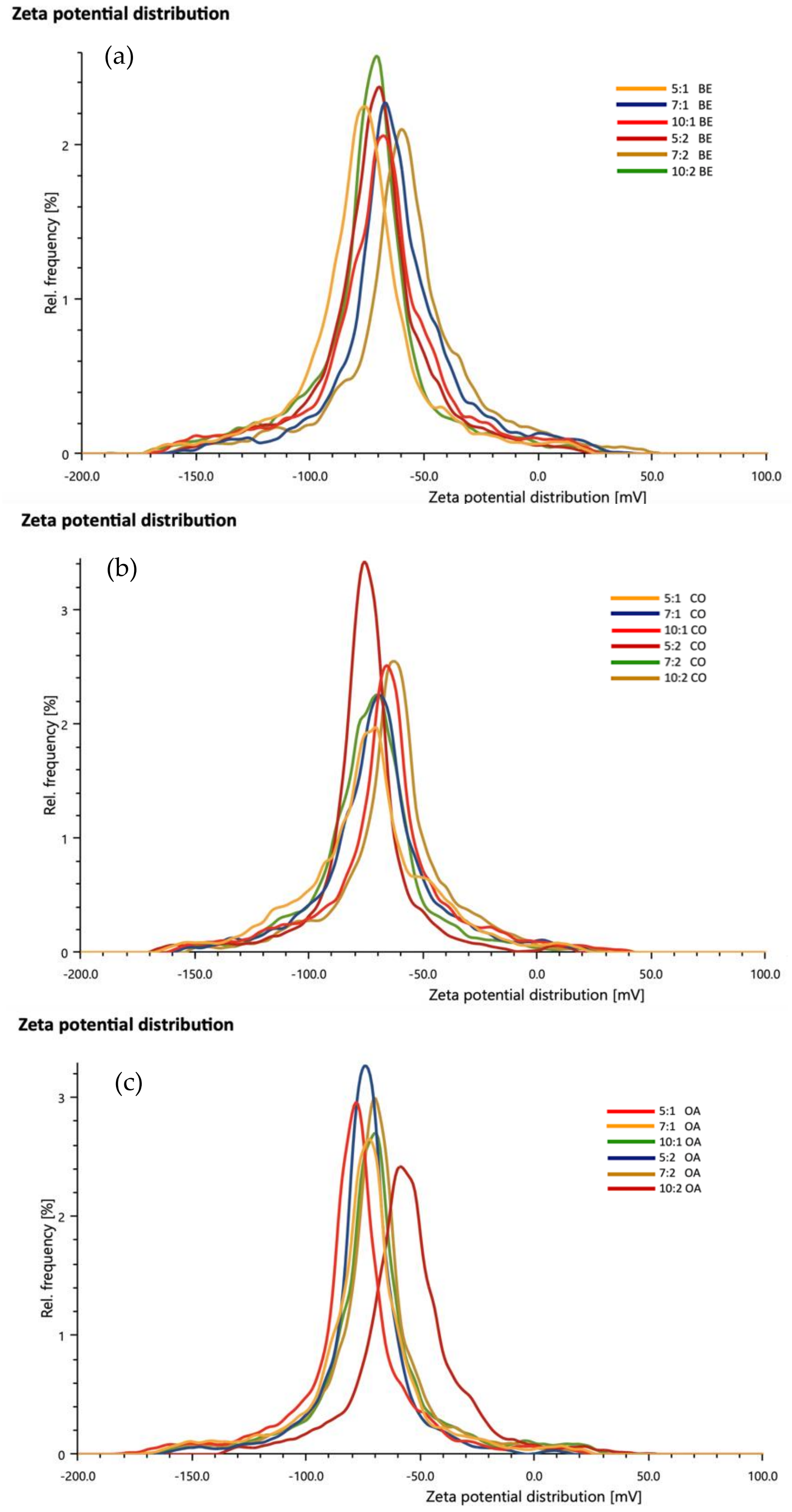

Figure S1. Zeta potential of lidocaine-loaded nanoemulsions formulated with (a) beeswax, (b) coconut oil and (c) oleic acid formulations.
